# OPEFB pretreatment using the low-cost *N*,*N*,*N*-dimethylbutylammonium hydrogen sulfate ionic liquid under varying conditions

**DOI:** 10.1038/s41598-023-48722-0

**Published:** 2023-12-15

**Authors:** S. M. Shahrul Nizan Shikh Zahari, Yichen Liu, Putian Yao, Mahfuzah Samirah Ideris, Hazeeq Hazwan Azman, Jason P. Hallett

**Affiliations:** 1 Department of Chemical Engineering, Faculty of Engineering, South Kensington Campus, Imperial College London, London, SW72AZ UK; 2 Industrial Chemical Technology Programme, Faculty of Science and Technology, Universiti Sains Islam Malaysia, Bandar Baru Nilai, 71800 Nilai, Negeri Sembilan Malaysia; 3 Key Laboratory of Green Chemistry and Technology, Ministry of Education, College of Chemistry, Sichuan University, 29 Wangjiang Road, Chengdu, 610064 Sichuan People’s Republic of China; 4 Centre for Foundation and General Studies, Universiti Selangor, Jalan Timur Tambahan, 45600 Bestari Jaya, Selangor Darul Ehsan Malaysia

**Keywords:** Biotechnology, Chemistry, Chemical engineering

## Abstract

This study investigates the effects of temperature and period on the pretreatment of OPEFB using the low-cost *N*,*N*,*N*-dimethylbutylammonium hydrogen sulfate ionic liquid ([DMBA][HSO_4_] IL) with 20 wt% of water. The results demonstrate that higher pretreatment temperatures (120, 150, and 170 °C) and longer periods (0.5, 1, and 2 h) enhanced lignin recovery, resulting in increased purity of the recovered pulp and subsequently enhanced glucose released during enzymatic hydrolysis. However, at 170 °C, prolonging the period led to cellulose degradation and the formation of pseudo-lignin deposited on the pulps, resulting in a decreasing-trend in glucose released. Finally, the analysis of extracted lignin reveals that increasing pretreatment severity intensified lignin depolymerisation and condensation, leading to a decrease in number average molecular weight (M_n_), weight average molecular weight (M_w_) and polydispersity index (Đ) values.

## Introduction

Oil palm empty fruit bunches (OPEFB), a by-product of palm oil processing, is a type of agricultural waste. Malaysia, the second-largest palm oil producer in the world, generated around 20 million metric tons of the waste in 2013. OPEFB is a cost-effective natural fibre that possesses good properties^1^.

The waste contains the following biopolymers: 40–50% cellulose, 20–30% hemicelluloses, and 15–20% lignin. The existence of these biopolymers allows OPEFB to be converted to multiple products in the form of energy, chemicals, and materials^1,2^. To convert OPEFB biopolymers into useful products, a pretreatment process is required to break linkages in the lignin–carbohydrate complexes, which connect the biopolymers. This enables the individual extraction and subsequent utilisation of each biopolymer in downstream processing^3–6^.

The use of protic ionic liquids (PILs) with hydrogen sulfate anion ([HSO_4_]^−^) in biomass pretreatment has gained significant attention. Synthesised through a simple acid–base neutralisation reaction between sulfuric acid (H_2_SO_4_) and an amine at room temperature with a 100% yield, these ILs have relatively low viscosity and high thermal stability over extended periods, making them suitable for industrial deployment^4,7^. More importantly, their ability to selectively solubilise lignin without dissolving cellulose is most effective at a water content of 20–40 wt%, which is ideal for biomass that generally contains 5–30 wt% moisture^8^. This approach is advantageous for some applications, such as paper and composites, that require the use of intact cellulose to achieve the desired mechanical and chemical properties^9–11^. Meanwhile, lignin can be converted into a variety of useful products, including carbon fibers and activated carbon^12–14^. Triethylammonium hydrogen sulfate ([TEA][HSO_4_]) is a common protic ILs that has been widely used in the pretreatment of various types of biomass, including OPEFB^4,7,15–21^. This IL, synthesised using inexpensive H_2_SO_4_, triethylamine (TEA) and water, can be made available at production cost as low as $0.78 kg^−1^, which is 80 times cheaper than the aprotic 1-butyl-3-methylimidazolium acetate ([BMIM][OAc])^8^.

*N,N,N*-Dimethylbutylammonium hydrogen sulfate ([DMBA][HSO_4_]), another low-cost protic ILs, has shown promising results in effectively removing lignin from various biomass sources, including coconut husks and shells^8^, miscanthus and pine softwood^22,23^. In a previous study, coconut husks and shells were pretreated with the IL under optimum conditions at 170 °C and 45 min, resulting in the removal of 77 wt% and 82 wt% of lignin, respectively^8^. The production price of this IL remains unreported in the chemical literature, but it is projected to be in the range of $1.24–$5.88 kg^−1^, consistent with other protic [HSO_4_]-based ILs^23^. The synthesis of [DMBA][HSO_4_], like [TEA][HSO_4_], proceeds via the neutralisation of dimethylbutylamine (DMBA) and H_2_SO_4_ at room temperature, producing the IL without needing purification and water being the only by-product. Technically, these features would make the IL synthesis to have a lower environmental impact as it reduces waste by-products, solvent losses, energy usage and CO_2_ generation. In contrast, there are 30 synthetic steps involved in producing 1-ethyl-3-methylimidazolium acetate ([EMIM][OAc]) with the consumption of many starting materials. As more starting materials are used, the environmental footprint increases^4^. The majority of alkylammonium [HSO_4_]-based ILs, including [DMBA][HSO_4_], are thermally stable, begin decomposing at 277 °C (compared to 215 °C for [EMIM][OAc]). This characteristic is advantageous for biomass pretreatment, typically conducted at 120–180 °C for extended periods, all of which are below the IL decomposition temperature. This means that the IL remains intact during the course of pretreatment, preventing the IL’s cation from undergoing sidechain dealkylation reactions, which are responsible for generating hazardous chemicals during pretreatment^23^.

Although [DMBA][HSO_4_] has been proven effective in pretreating various types of biomass, its efficacy in the pretreatment of OPEFB has yet to be explored. Given its success with other biomass types, it can therefore be assumed that the IL could be effective for the pretreatment of OPEFB. Based on this assumption, this study conducted pretreatment OPEFB pellets with [DMBA][HSO_4_] containing 20 wt% and investigated the influence of different temperatures (120, 150 and 170 °C) and periods (0.5, 1, and 2 h) upon: purity of cellulose pulp, degree of delignification and structural alterations of lignin. For the effect of temperature, 120 °C was chosen as the lower limit based on its demonstrated efficacy in lignin removal over an extended period as reported previously. Temperatures 150 °C and 170 °C were selected to increase the pretreatment effectiveness.

## Methodology

### Materials and equipment

Chemical precursors were purchased from the Sigma-Aldrich, otherwise stated. ^1^H- and ^13^C-NMR were recorded on a Bruker 400 MHz spectrometer. Chemical shifts (δ) are reported in ppm, the DMSO signal at 2.500 (^1^H dimension) and 39.520 (^13^C dimension). The acid-to-base (A/B) ratio of the IL was measured using a Karl-Fisher titrator (V20 volumetric Titrator-Mettler-Toledo) and the analytical balance a Sartorius CPA 1003-S balance (± 0.001 g).

### Synthesis of [DMBA][HSO4]

*N,N,N*-Dimethylbutylamine (446 g, 3.5 mol) was cooled in an ice bath. Under vigorous stirring, an aqueous solution of sulfuric acid (3.5 mol) was added dropwise to the amine. Excess water was then removed from the resultant ionic liquid by a rotary evaporator.

^1^H-NMR: δH (400 MHz, DMSO-d6)/ppm: ^13^C-NMR: δC (101 MHz, DMSO-d6)/ppm: 46.21; A/B ratio: 1.02 ± 0.004.

### Biomass feedstock

Oil palm empty fruit bunches (OPEFB) as pellets were donated by Eureka Synergy Biomass Sdn. Bhd., Malaysia. After drying at room temperature for several days, the pellets were manually disintegrated into smaller pieces and later kept in a sealed bag. Using the published procedure by NREL^24^, the biomass was found to contain 11.07 ± 0.26% of moisture.

### OPEFB pretreatment

**Figure 1:**
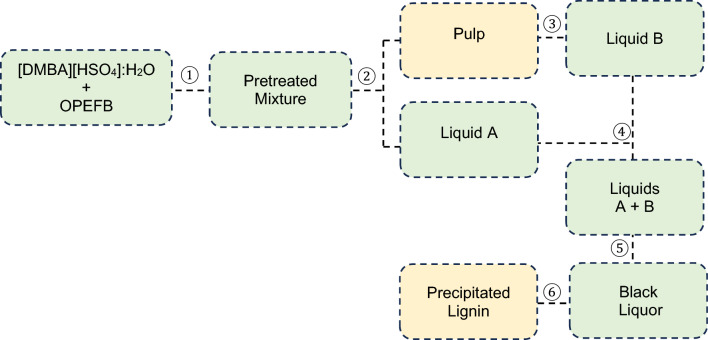
OPEFB pretreatment with 80 wt% [DMBA][HSO_4_] and 20 wt% water at a 1:10 biomass-to-solvent ratio.

The protocol is summarised in Fig. 1. In an ACE pressure tube, OPEFB (2 g, oven-dried basis) was mixed with [DMBA][HSO_4_] (16 g) and distilled water (4 g), yielding a ratio of 1:10 (biomass-to-solvent). After mixing the content using an orbital shaker, the tube was placed in a convection oven and incubated at various temperatures and periods (Step 1). After cooling the tube to room temperature, the pretreatment mixture was diluted with ethanol (EtOH) (40 mL) and then centrifuged at 3000 r.p.m. The liquid solution, labelled as ‘Liquid A’, was discarded, and stored for lignin recovery (Step 2). The undissolved solid, named as ‘pulp’, was continuously purified with EtOH in a Soxhlet extractor overnight and later air-dried at room temperature. The used EtOH was collected and labelled as ‘Liquid B’ (Step 3).

For lignin recovery, the Liquid A was mixed with the Liquid B (Step 4). The mixture was concentrated by evaporating EtOH on a rotary evaporator, yielding a black liquor (Step 5). Distilled water (40 mL) was then added to the liquor, and the mixture was centrifuged at 3000 r.p.m. After discarding the liquid, the lignin was recovered as a black precipitate, which was later freeze dried overnight (Step 6).

### Biomass and pulp characterisation

Moisture content and compositional analysis of raw EFB and recovered pulps were determined according to the NREL protocol ‘Determination of Structural Carbohydrates and Lignin in Biomass by the NREL’^24^. Enzymatic saccharification was conducted according to the Low Solids Enzymatic Saccharification of Lignocellulosic Biomass protocol published by the NREL^25^.

### Gel permeation chromatography (GPC)

GPC was used to determine the differences of molecular weight of lignin recovered following the pretreatment process. For each measurement, 15–20 mg was dissolved in DMF containing 0.1% LiBr. The resultant solution was filtered through a 0.22 μm nylon filter and analysed on a Shimadzu equipped with a photodiode array and refractive index detectors. Polystyrene standards were used to calibrate the instrument.

### 2D-HSQC NMR spectroscopy

Two dimensional ^1^H and ^13^C heteronuclear single-quantum coherence (HSQC) NMR of the recovered lignin samples were measured. For each measurement, 15–20 mg of the lignin was dissolved in 0.75 mL of DMSO-d^6^ and introduced into an NMR tube. The analysis was performed on a Bruker Avance III HD 800 MHz equipped with triple resonance cryoprobes. The spectral width of 10 ppm in F2 (^1^H) with 2048 data points and 160 ppm in F1 (13C) with 256 data points was used, and interscan delay was set at 1.5 s. For each sample, a one-hour measurement was carried out by using 8 scans. The spectra were analysed by MestReNova software (14.0.0). To measure the condensation of guaiacyl (G) and syringyl (S) units, the signals for G_2_, G_2cond._, S_2,6_, and S_2,6cond_. were integrated taking the same areas for all samples.

The integrals were normalized to the abundance of G_2_ and G_2cond._ to quantitatively determine the degree of condensation of lignin. Chemical shifts were reported in ppm and referenced to DMSO-d^6^, (δH 2.50, δC 39.50).

## Results and discussion

### Pulp and lignin recovery

**Figure 2:**
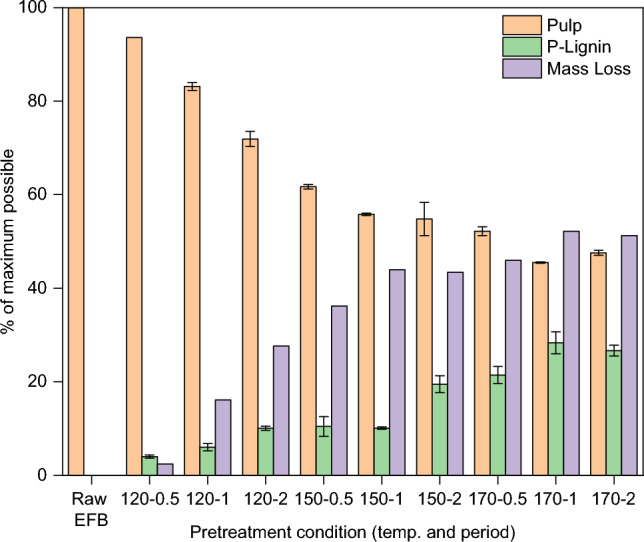
Pulp, precipitated lignin, and mass loss after pretreatment of OPEFB with 80 wt% [DMBA][HSO_4_] and 20 wt% water at a 1:10 biomass-to-solvent ratio under different conditions. The mass loss is the matter that dissolved in the IL that could not be recovered. Error bars are standard deviations of triplicate samples.

Figure 2 shows the recovery of pulp and lignin as well as the mass loss. Overall, the pretreatment using [DMBA][HSO_4_] decreased the pulp recovery and simultaneously increased the lignin recovery after aqueous precipitation. This proved the ability of the IL to break down lignin–carbohydrate complexes (LCCs) in OPEFB and concurrently extract the lignin. As a result, pulp and lignin were obtained as two individual solids. Generally, higher pretreatment severity (longer period or higher temperature) yields a much lesser pulp due to a much higher lignin extraction by the IL^19^. This is consistent with our results. At 120 °C, the recovery of lignin increased by 60% when the period was prolonged from 0.5 to 2 h.

For the pretreatment at 150 °C, the pulp recovery expectedly decreased as period extended from 0.5 to 2 h. In contrast, the lignin recovery remained constant at ca. 10% for 0.5–1 h-period and then increased by 50% in the subsequent 2 h-period. One plausible explanation for the constant lignin recovery from 0.5 to 1 h is that the lignin extracted at 1 h-period might have contained smaller lignin fragments than those extracted at 0.5 h, which could not be precipitated by water during the precipitation step (see Fig. 1, Step 6). The formation of smaller lignin fragments over time during the pretreatment of biomass utilising [HSO_4_]-based ILs has been reported in previous studies, attributed to increased cleavage of aryl ether bonds in the polymer^8,15,17–19^. The 50% increase in lignin recovery for the 2 h-period is indicative of increased disruption of lignin–carbohydrate complexes (LCCs) in OPEFB.

A different pattern was observed at 170 °C. Within 0.5–1 h, the material removal was gradually increased, yielding a much higher lignin recovery relative to pulp recovery. After 2 h, there was a small decrease in the amount of lignin and a slight increase in the amount pulp. This may be a sign of overtreatment. Overtreatment promotes lignin depolymerisation, producing more water-soluble fragments that cannot be precipitated by water, leading to decreased lignin recovery. Additionally, overtreatment can also cause lignin fragment condensation and redeposition and/or formation of pseudo-lignin on the pulp, contributing to increased pulp weight^19^.

The exact mechanism underlying the formation of pseudo-lignin during pretreatment of biomass using acid-bearing ionic liquids remains unclear. This is because the material can be formed through various consecutive reactions (degradation, cross-linking and re-condensation) from biopolymers (cellulose, hemicellulose and lignin), their intermediates, hydroxylmethylfurfural (HMF) and furfural^26^. Hu et al*.*^27^ pretreated cellulose and holocellulose using a dilute acid. These carbohydrates depolymerised into sugars, which subsequently degraded and re-polymerised to form pseudo-lignin^27^. Another study, investigating the formation of pseudo-lignin during pretreatment in the presence of [TEA][HSO_4_], proposed that pseudo-lignin formation was due to the condensation and re-polymerisation of lignin fragments, carbohydrates, and their intermediates^26^. In this work, [DMBA][HSO_4_] also provided an acidic environment; therefore, the pseudo-lignin was speculated to be produced under a similar mechanism to that reported in the presence of [TEA][HSO_4_]^26^. Further investigations are required to unveil the mechanism and structures of pseudo-lignin formation in the context of pretreatment of OPEFB utilising [DMBA][HSO_4_].

### Compositional analysis of pulps

**Figure 3:**
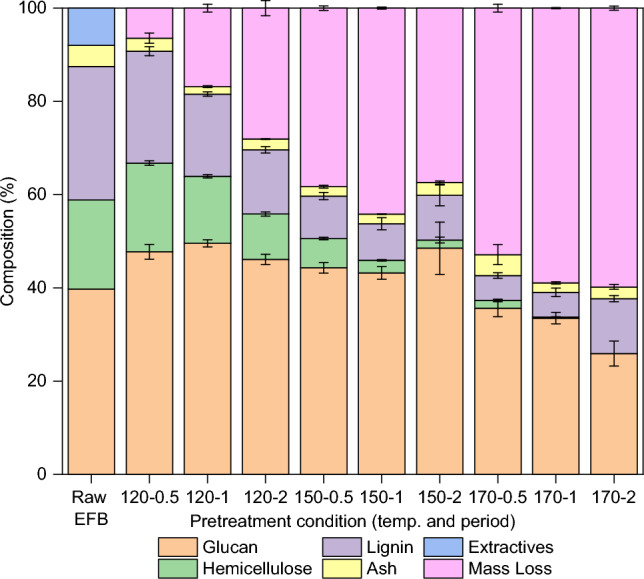
Glucan, hemicellulose, and lignin content of untreated OPEFB and pulps recovered via pretreatment of OPEFB with 80 wt% [DMBA][HSO_4_] and 20 wt% water at a 1:10 biomass-to-solvent ratio under different conditions. The mass loss is the matter that dissolved into the ionic liquid. Error bars are standard deviations of triplicate samples.

The composition of untreated OPEFB and recovered pulps was analysed and the results are summarised in Fig. 3. The removal of hemicellulose occurred rapidly at all three studied temperatures with prolonged periods and was complete at 170 °C after 2 h.

At 120 °C, both lignin and hemicellulose removal showed an increasing trend as the period extended from 0.5 to 2 h. However, the applied conditions appeared to be less effective, retaining ca. 55% hemicellulose and lignin in the pulp obtained at the longest period of 2 h (relative to raw OPEFB).

At 150 °C, hemicellulose removal gradually increased over period. Conversely, the lignin content initially decreased within 0.5–1 h but then increased as the period was prolonged to 2 h. Pretreatment at 170 °C led to further hemicellulose removal with prolonged periods; however, the conditions also caused a gradual decrease in cellulose content. Unlike cellulose and hemicellulose, pretreatment at 170 °C for 0.5–1 h removed more than 80% lignin relative to that in raw OPEFB. However, further extending the period to 2 h increased lignin content in the recovered pulp by ca. 50% compared to those obtained at 0.5 h and 1 h.

The results in Figs. 2 and 3 show a direct association. Increasing pretreatment severity (higher temperature and longer period) led to a gradual increase in mass loss (Fig. 2), which can be directly linked to the extensive removal of hemicellulose from the pulp (Fig. 3). Hemicellulose is known to readily degrade under acidic conditions and elevated temperatures, yielding water-miscible 5-hydroxylmethyl furfural (HMF) and furfural. In this context, it was believed that the IL played a dual role by removing hemicellulose from the pulp and at the same time catalysing the polymer degradation to furfural catalysed through its [HSO_4_]^−^ anions. As depicted in Fig. 3, cellulose content in the pulp decreased during pretreatment at 170 °C over time, which is consistent with the decreasing-trend observed in the pulp recovery (Fig. 2). This can be attributed to the hydrolysis of amorphous cellulose, producing low degree of polymerisation (DP) cellulose that dissolved in IL/H_2_O mixture. This is supported by the fact that amorphous cellulose is easier to hydrolyse as water and protons can more easily penetrate into amorphous regions, leading to the cleavage of the β-1,4-glycosidic bonds^18^.

From the compositional analysis in Fig. 3, the pulp recovered at 170 °C for 2 h had a lignin content twice as high as that found in the pulps recovered after 0.5 h and 1 h-pretreatment. This contradicts the expected trend of decreasing lignin content in the pulp with increasing pretreatment severity.

The unexpected increased lignin content could be linked to the formation of condensed higher molecular weight ‘pseudo-lignin’, an acid insoluble organic matter that deposits on the pulp surface. Consequently, there will be a slight increase in the percentage of pulp recovery, as observed in the comparison of the pulps recovered after 2 h against 1 h-pretreatment. This occurs because pseudo-lignin is insoluble in acidic solutions, making the compound unable to be separated from the pulp. This is a reason why it is difficult to distinguish between ‘true’ lignin and pseudo-lignin by the compositional analysis^18^.

A similar observation was previously reported for the pretreatment of Miscanthus with [TEA][HSO_4_] during an extended period^18^. Extensive discussions regarding the plausible mechanisms of the formation of pseudo-linin in [HSO_4_]-based ILs have been reported in the chemical literature^15,18,19^.

### Glucose release via enzymatic hydrolysis

**Figure 4:**
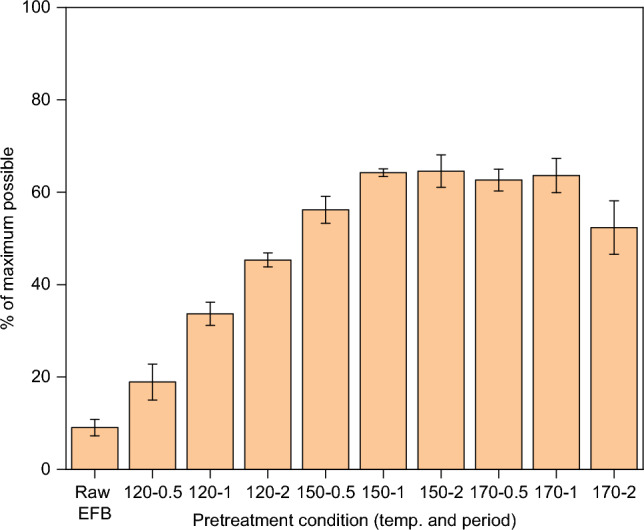
Glucose released by untreated OPEFB and recovered pulps by enzymatic hydrolysis at 50 °C for 72 h. Error bars are standard deviations of triplicate samples.

The glucose yields after 72 h of enzymatic hydrolysis are shown in Fig. 4. Even without pretreatment, untreated OPEFB released ca. 9% of glucose, which could be due to the enzyme’s preferential actions at the reducing ends (RE) and nonreducing ends (NRE) of cellulose chains that are more accessible. It has been demonstrated that glucose yield obtained through enzymatic hydrolysis is highly dependent on the lignin removal^19,28^. In other words, the more the lignin is removed, the higher the release of glucose from the pulp. This is because lignin in the biomass/pulp inhibits the productive binding of cellulase enzymes to cellulose, thus lowering the glucose yield^19^.

**Figure 5:**
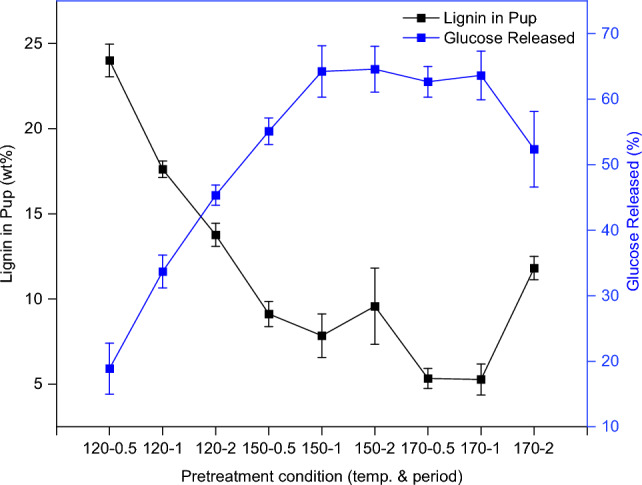
The dependence of glucose release with lignin removal of pulps recovered from pretreatment recovered via pretreatment of OPEFB with 80 wt% [DMBA][HSO_4_] and 20 wt% water at a 1:10 biomass-to-solvent ratio under different conditions. Error bars are standard deviations of triplicate samples.

Overall, the dependence of glucose release with lignin removal was evidenced in this study. As pretreatment severity increased, with higher temperature and longer period, the glucose yield for most conditions showed an increasing trend due to a corresponding increase in the removal of lignin (shown in Fig. 5).

For the pretreatment at 150 °C, the pulps recovered after 0.5 h and 1 h showed the anticipated inverse relationship between lignin content and glucose yield. Surprisingly, the 2 h-pulp produced a comparable glucose yield to that of the 1 h-pulp (64%), despite containing about ca. 18% more lignin. In theory, the presence of lignin and hemicellulose impedes the enzymatic conversion of cellulose to glucose by restricting the access enzymes molecules to cellulose substrate. Therefore, the removal of these biopolymers is necessary to enhance the structural openness and accessibility of cellulose to enzyme molecules^29^. From the compositional analysis in Fig. 3, the 2 h-pulp had the lowest hemicellulose content, being 1.5 times lower than the 1 h-pulp and 3.5 times lower than the 0.5 h-pulp. This aligns with the aforementioned theory, indicating that the increased glucose yield for 2 h-pulp is a result of reduced hemicellulose content. Regarding the lignin content, it is plausible that the additional lignin content in the 2 h-pulp (compared to 1 h-pulp) is mainly a physical presence rather than being chemically bound. This type of lignin may not significantly interfere with the accessibility of cellulose substrate by the enzyme molecules. We are currently investigating this aspect.

At 170 °C, the recovered pulp after 2 h-pretreatment produced a much lower glucose yield compared to the pulps recovered after 0.5 h and 1 h-pretreatment. This is expected as the pulp obtained after 2 h contained two times more lignin that those recovered after 0.5 h and 1 h-pretreatment (Fig. 5) The presence of pseudo-lignin, as indicated by the additional 50% lignin content, could be an additional reason to the decreased glucose yield produced by the pulp of 2 h-pretreatment. Like the ‘true’ lignin, pseudo-lignin deposited on the surface blocks enzyme access to cellulose. It also has the tendency to unproductively bind to the cellulases enzymes, further impeding the enzymatic hydrolysis of cellulose to glucose^5,29^.

### Lignin characterisation

#### 2D HSQC-NMR

**Figure 6:**
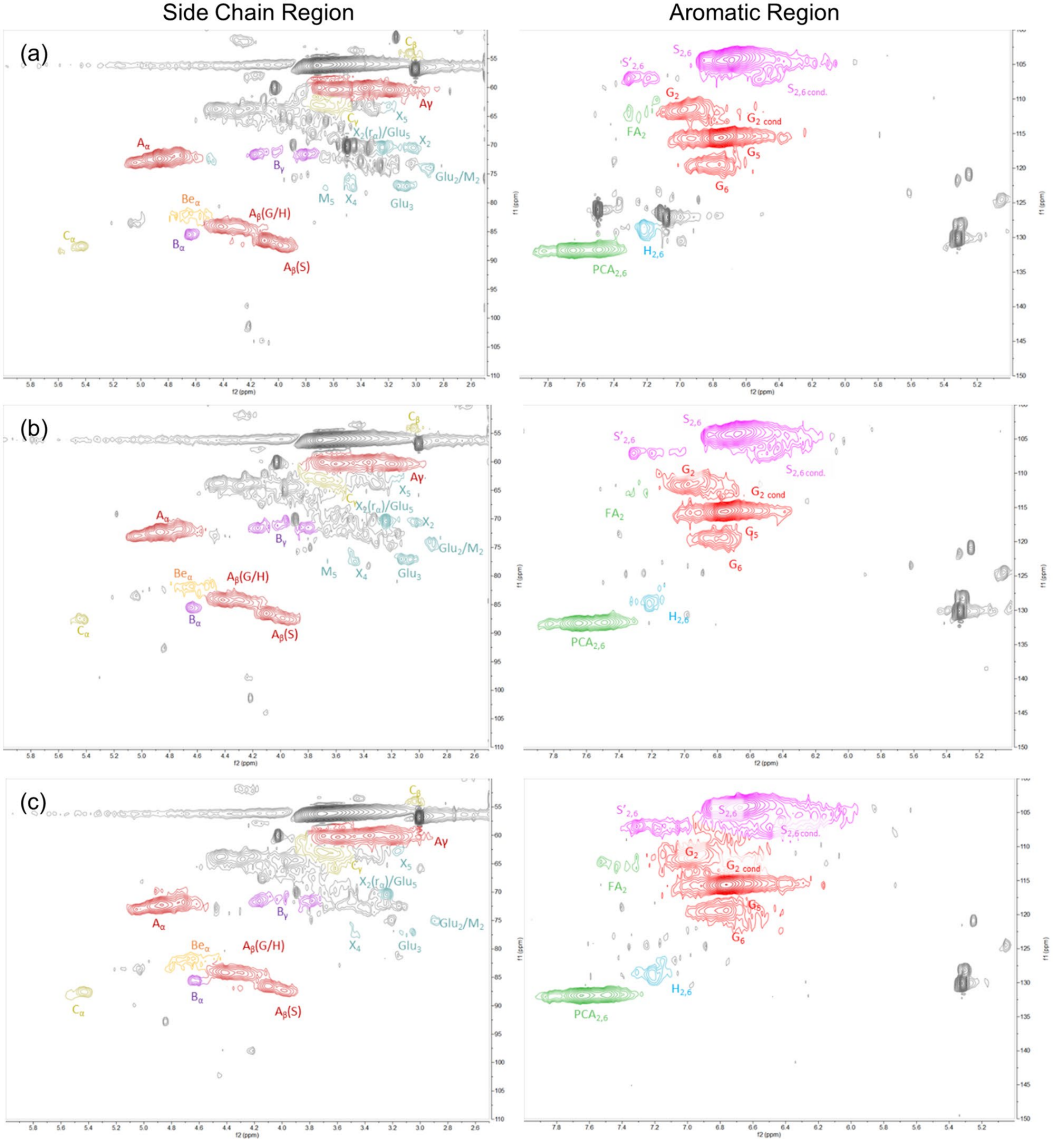
2D-HSQC-NMR spectra of extracted lignin from pretreatment of OPEFB at 120 °C, with the sidechain and aromatic regions for different pretreatment periods: 0.5 h (**a**), 1 h (**b**), and 2 h (**c**).

This analysis estimates the subunit composition, the type of linkages, the degree of condensation and the presence of ether bonds^18^. Figure 6 shows HSQC spectra of lignins obtained at 120 °C, illustrating cross-signals appeared in side-chain (d_C_/d_H_ 40–110/2.5–6.0) and aromatic (d_C_/d_H_ 100–150/5.0–8.0) regions. The spectra of other lignins recovered at 150 °C and 170 °C can be found in Fig. S1 and S2 in ESI. The cross-signals appeared in all recorded HSQC spectra were compared with those published literature^30–34^, leading to the assignment of lignin linkages and subunits as summarised in Table 1.

**Table 1:** Assignments of cross-signals for the recorded 2D-HSQC-NMR spectra of precipitated lignins^30–34^.

Label	δ_H_/δ_C_ (ppm)	Assignment
OMe	56.0/3.74	C–H in phenols or methoxy
Glu2	74.8/2.88	C2/H2 in β-D-glucopyranoside
Glu3	76.7/3.06	C3/H3 in β-D-glucopyranoside
Glu5	70.3/3.18	C5/H5 in β-D-glucopyranoside
X2	72.6/3.02	C2/H2 in β-D-xylopyranoside
X2(r_α_)	69.7/3.24	C_2_/H_2_ in β-D-xylopyranoside of xylans reducing end
X4	75.3/3.45	C4/H4 in β-D-xylopyranoside
X5	62.9/3.13 or 3.78	C5/H5 in β-D-xylopyranoside
M5	76.9/3.60	C5/H5 in β-D-mannopyranoside
Be_α_	82.0/4.65	Cα–Hα in benzyl ether LCC structures
Aα	71.8/4.86	Cα–Hα in β-O-4′ (A) and γ-acetylated β-O-4′
Aβ(G/H)	83.9/4.28	Cβ–Hβ in β-O-4′ (A) linked to G and H
Aβ(S)	85.9/4.12	Cβ–Hβ in β-O-4′ (A) linked to S
Aγ	59.8/3.61	Cγ–Hγ in β-O-4′ (A)
Bα	84.8/4.67	Cα-Hα in β-β′ and α-O-γ′ resinol (B)
Bβ	53.5/3.06	Cβ–Hβ in β-β′ and α-O-γ′ resinol (B)
Bγ	71.0/3.82–4.20	Cγ–Hγ in β-β′ and α-O-γ′ resinol (B)
Cα	86.8/5.46	Cα–Hα in β-5′ and α-O-4′ phenylcoumaran (C)
Cβ	53.7/3.12	Cβ–Hβ in β-5′ and α-O-4′ phenylcoumaran (C)
Cγ	62.5/3.73	Cγ–Hγ in β-5′ and α-O-4′ phenylcoumaran (C)
S2,6	104.3/6.71	C2–H2 and C6–H6 in syringyl (S)
S’2,6	106.5/7.29–7.17	C2–H2 and C6–H6 in syringyl (Oxidized α-ketone) (S)
H3,5	114.2/6.68	C3–H3 and C5–H5 in *p*-hydroxyphenyl(H)
H2,6	128.2/7.19	C2-H2 and C6-H6 in *p*-hydroxyphenyl (H)
G2	111.8/6.98	C2–H2 in guaiacyl (G)
G5	115.3/6.75 and 6.93	C5–H5 in guaiacyl (G)
G6	119.3/6.90	C6–H6 in guaiacyl (G)
PCA3,5	115.5/6.77	C3–H3 and C5–H5 in *p*-coumarate (PCA)
PCA2,6	130.1/7.45	C2–H2 and C6–H6 in *p*-coumarate (PCA)
FA2	111.0/7.32	C2–H2 in ferulate (FA)

The samples were recovered via pretreatment of OPEFB with 80 wt% [DMBA][HSO_4_] and 20 wt% water at a 1:10 biomass-to-solvent ratio under different conditions.

**Figure 7:**
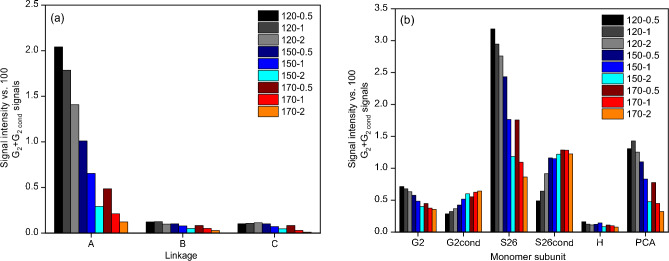
(**a**) chemical linkages detected (A: β-Ο-4, B: β-β, and C:α-Ο-4) in the side-chain region and (**b**) monomer subunits detected in aromatic region in 2D-HSQR NMR spectra of the precipitated lignins. The samples were obtained from pretreatment of OPEFB with 80 wt% [DMBA][HSO_4_] and 20 wt% water at a 1:10 biomass-to-solvent ratio under different conditions.

Figure 6 (as well as Fig. S1 and S2 in ESI) demonstrates that the extracted lignin from OPEFB composed of guaiacyl (G, FA), syringyl (S, S′), and *p*-hydroxyl-phenol (H, PCA) subunits connected by β-Ο-4 (A), β-β (B) and α-Ο-4 (C) linkages, as indicated by the cross-signals emerged in the aromatic and the side-chain regions, respectively, while the aromatic region displayed type of lignin subunits. Figure 7 summarises the abundance of linkages and subunits obtained using volume peak integration.

#### Lignin–carbohydrate complexes (LCCs)

**Figure 8:**
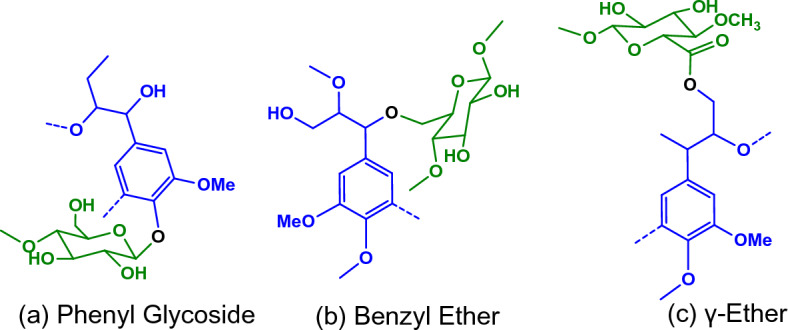
Proposed structures of lignin carbohydrate complexes (LCCs) bonds in OPEFB^35^.

Understanding the nature of LCCs in OPEFB is important because the LCCs are a major contributor to the recalcitrance of the biomass. These LCCs within biomass typically comprise phenyl glycoside, benzyl ether, and γ-ether structures, as illustrated in Fig. 8a–c. The side chain regions in (along with Fig. S1 and S2 in ESI) displayed several cross-signals suggesting β-D-glucopyranoside (G), β-D-xylopyranoside (X), and β-D-mannopyranoside (M) sugars, all of which are hemicellulose monomers and components of LCCs. A cross-signal detected δ_C_/δ_H_ = 82.0/4.65 was assigned to the presence of a-benzyl ether (BEa) bonds, which link the hemicellulose sugars to lignin as proposed by the chemical structure in Fig. 8a. The BEa cross-signal was initially quite intense for all lignins extracted at 120 °C, but its intensity decreased as the pretreatment severity increased. The BEa cross-signal completely disappeared in lignin extracted at 170 °C for 2 h, as shown in Fig. S2 in ESI. This is expected because ether bonds are resistant to hydrolysis under mild conditions. However, as the pretreatment severity increases, the hydrolysis rate of ether bonds increases.

Interestingly, the aromatic region in Fig. 6 (along with Fig. S1 and S2 in ESI) exhibited the presence of *p*-coumaric acid (PCA) at relatively higher quantities, as summarised in Fig. 7b. This finding suggests another possible bond structure of LCCs in OPEFB through esterification of PCA with the hydroxyl groups of hemicellulose sugars, as proposed by the chemical structure in Fig. 8b.

The proportion of PCA steadily decreased with increasing temperature and period, indicating increased hydrolysis of ester bonds catalysed by [HSO_4_]^−^ ions that eventually removed hemicellulose sugars.

#### Linkages in lignin structure

As shown in Fig. 7a, β-Ο-4 was the predominant linkage detected in lignins extracted from OPEFB. This linkage has been reported to have the highest content in the lignin extracted from lignocellulosic biomass^36^. In contrast, β-β and α-Ο-4 linkages were found in minor quantities. Prolonging the pretreatment period at 120 °C caused a rapid decrease in the content of β-Ο-4 linkages, while the contents of β-β and α-Ο-4 appeared unchanged. This demonstrates that the β-Ο-4 linkage is the most susceptible to chemical and thermal degradation, consistent with previous research^36^. For pretreatments at 150 °C and 170 °C, the content of all linkages (β-Ο-4, β-β and α-Ο-4) gradually decreased with prolonging the period mostly likely due to intensified hydrolysis reaction catalysed by [HSO_4_]^−^ anions.

#### Subunits in lignin structure

**Figure 9:**
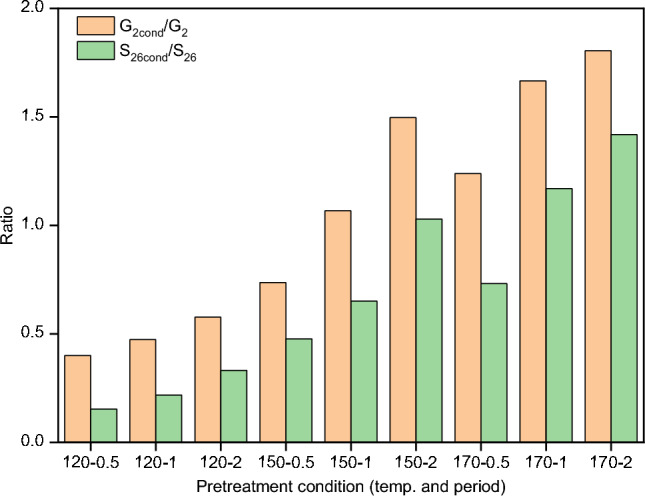
Calculated ratios of the integration of cross-signals of G_2-cond_/G_2_ and S_2,6-cond_/S_2,6_.

Figure 7b reveals all lignins were highest in S subunits (S_2,6_ and S_2,6-cond_) and lowest in H subunits. In Fig. 9, increasing pretreatment severity (higher temperature and longer period) resulted in an increasing trend of both G_2cond_/G_2_ and S_2,6cond_/S_2,6_ ratios. As an example, prolonging the pretreatment period from 0.5 to 2 h at 150 °C exhibited a two-fold increase in G_2cond_/G_2_ and S_2,6cond_/S_2,6_ ratios. Meanwhile, elevating the pretreatment temperature from 120 to 170 °C (within 0.5 h) led to 3–5 times increase in both G_2cond_/G_2_ and S_2,6cond_/S_2,6_ ratios. This observation strongly indicates that lignin condensation was promoted under more severe pretreatment conditions. Figure 9 also shows that the ratios of S_2,6cond_/S_2,6_ are notably lower compared to those of G_2cond_/G_2_. This finding is in line with the anticipated higher steric hindrance of the S unit than the G unit, resulting in reduced reactivity of the S unit^37^.

#### Molar mass

Lignins, isolated under different pretreatment conditions, were analysed by GPC (profiles are shown in Fig. S3 in the ESI). Table 2 summarises number average molecular weight (M_n_), weight average molecular weight (M_w_) and polydispersity index (Đ) values. Prolonged pretreatment at 120 °C from 0.5 to 1 h led to a reduction in M_w_ (by ca. 50%) and its corresponding M_n_ and Đ. However, further extending the pretreatment to 2 h resulted in a slight increase in M_w_ and its corresponding M_n_ and Đ compared to 1 h-pretreatment. It should be noted that our pretreatment extracted lignin through dissolving the polymer in [DMBA][HSO_4_] over the process period.

**Table 2:** Molar mass of precipitated lignins isolated from pretreatment of OPEFB with 80 wt% [DMBA][HSO_4_] and 20 wt% water at a 1:10 biomass-to-solvent ratio under different conditions.

Sample	M_n_ (D_a_)	M_w_ (D_a_)	Đ
120-0.5	15,507	132,673	8.56
120-1	13,000	55,615	4.28
120-2	13,928	59,189	4.25
150-0.5	23,108	411,881	17.82
150-1	51,383	971,250	18.90
150-2	14,097	207,134	14.69
170-0.5	21,227	296,425	13.96
170-1	14,462	129,638	8.96
170-2	12,044	46,276	3.84

We therefore believe that the decrease in M_w_ observed during 0.5–1 h-pretreatment is likely due to the depolymerisation of the dissolved lignin, consequently decreasing molecular weight of lignin. Meanwhile, a slight increase in M_w_ observed in 2 h-pretreatment could indicate cross-condensation of smaller lignin fragments, eventually forming much larger molecules. This is further corroborated by the increased G_2-cond_/G_2_ and S_2,6-cond_/S_2,6_ ratios, as shown in Fig. 7b.

For lignins obtained at 150 °C, the M_n_ value initially increased twofold upon prolonging the pretreatment period from 0.5 to 1 h, likely indicating increased extraction of longer lignin chains. However, further extending the pretreatment period to 2 h resulted in a significant decrease in M_n_ value, suggesting the occurrence of lignin depolymerisation.

In contrast, lignins obtained from pretreatment at 170 °C showed a decreasing trend in M_w_ with its corresponding M_n_ and Đ over time, suggesting that the lignin depolymerisation was favoured over lignin cross-condensation at this temperature. It was worth noticing that the molecular weights of the lignin obtained from the pretreatments at 120, 150, and 170 °C showed different trends with the increase of reaction time, suggesting that lignin underwent depolymerisation followed by condensation reactions. The results of 2D-HSQC (Fig. 7b) lend further supports, showing an increase in G_2-cond_/G_2_ and S_2,6-cond_/S_2,6_ ratios, implying an increase in the extent of lignin condensation with prolonging the pretreatment period. Overall, the GPC analysis appears to conclude that the pretreatment severity influences the competition between depolymerisation and condensation of lignin.

## Conclusion

This study explores the use of [DMBA][HSO_4_] IL as a Brønsted acid catalyst and a delignifying agent in separating lignin from cellulose-rich pulps in OPEFB via a pretreatment process. Pretreatment temperatures (120, 150, and 170 °C) and periods (0.5, 1, and 2 h) were varied to examine their impacts on lignin recovery, pulp purity, and the physical and chemical characteristics of the extracted lignin.

The findings demonstrate that the IL, in the presence of 20 wt% of water, effectively disrupted lignin–carbohydrate complexes (LCCs), enabling the recovery of lignin and pulps as separate solids.

As the pretreatment severity intensified (higher temperature and longer period), the study observed an increase in lignin recovery and a simultaneous decrease in pulp recovery. Additionally, the purity of the pulp also increased, as indicated by the decreased lignin and hemicellulose content. However, at 170 °C, extending pretreatment period resulted in cellulose degradation and an increasing trend in lignin content. The unexpected increased in lignin content, indicative of the presence of pseudo-lignin in the pulps, negatively impacted the glucose released via enzymatic hydrolysis. Finally, the analysis of extracted lignin reveals that increasing pretreatment severity intensified lignin condensation reaction, reducing the corresponding number average molecular weight (M_n_), weight average molecular weight (M_w_) and polydispersity index (Đ).

## Supplementary Information

Supplementary Figures.

## Data Availability

All data acquired in this study are presented in this published article and its supplementary information documents. Requests for additional data or materials from this study can be directed to the corresponding author, Dr S.M.S.N. Shikh Zahari via email at shahrul.zahari@usim.edu.my.
