# Author Correction: OPEFB pretreatment using the low-cost *N*,*N*,*N*-dimethylbutylammonium hydrogen sulfate ionic liquid under varying conditions

**DOI:** 10.1038/s41598-025-88935-z

**Published:** 2025-02-18

**Authors:** S. M. Shahrul Nizan Shikh Zahari, Yichen Liu, Putian Yao, Mahfuzah Samirah Ideris, Hazeeq Hazwan Azman, Jason P. Hallett

**Affiliations:** 1 Department of Chemical Engineering, Faculty of Engineering, South Kensington Campus, Imperial College London, London, SW72AZ UK; 2 Industrial Chemical Technology Programme, Faculty of Science and Technology, Universiti Sains Islam Malaysia, Bandar Baru Nilai, 71800 Nilai, Negeri Sembilan Malaysia; 3 Key Laboratory of Green Chemistry and Technology, Ministry of Education, College of Chemistry, Sichuan University, 29 Wangjiang Road, Chengdu, 610064 Sichuan People’s Republic of China; 4 Centre for Foundation and General Studies, Universiti Selangor, Jalan Timur Tambahan, 45600 Bestari Jaya, Selangor Darul Ehsan Malaysia

Correction to: *Scientific Reports* 10.1038/s41598-023-48722-0, published online 15 December 2023

The Acknowledgements section in the original version of this Article contained errors. The corrected Acknowledgements section now reads:

“S.M.S.N. Shikh Zahari acknowledges the Ministry of Higher Education of Malaysia (MOHE) for the research fund under the Fundamental Research Grant Scheme for Research Acculturation of Early Career Researcher (FRGS-RACER) with reference number RACER/1/2019/TK05/USIM//1. He also wishes to acknowledge MOHE and Universiti Sains Islam Malaysia (USIM) for the scholarship (KPT(BS)860407) to pursue postdoctoral research fellow at Imperial College London, UK. All authors would like to thank Dr. Roberto Rinaldi from the Department of Chemical Engineering, Imperial College London, for granting access to GPC instrument in his laboratory. They also acknowledge Mr. Danny Ho of Eureka Synergy Biomass Sdn. Bhd., Malaysia for supplying OPEFB pellets.”

The original Article has been corrected.

