# Supplementary material for: OPEFB pretreatment using the low-cost N,N,N-dimethylbutylammonium hydrogen sulfate ionic liquid under varying conditions
Source: Sci Rep. 2023 Dec 15;13:22354. doi: 10.1038/s41598-023-48722-0 (PMC10724162; doi:10.1038/s41598-023-48722-0)
Supplement: Supplementary file 1 — Supplementary Figures. [file 41598_2023_48722_MOESM1_ESM.docx]

Electronic supplementary information (ESI)

OPEFB pretreatment using the low-cost N,N,N-dimethylbutylammonium hydrogen sulfate ionic liquid under varying conditions

Side-Chain Region


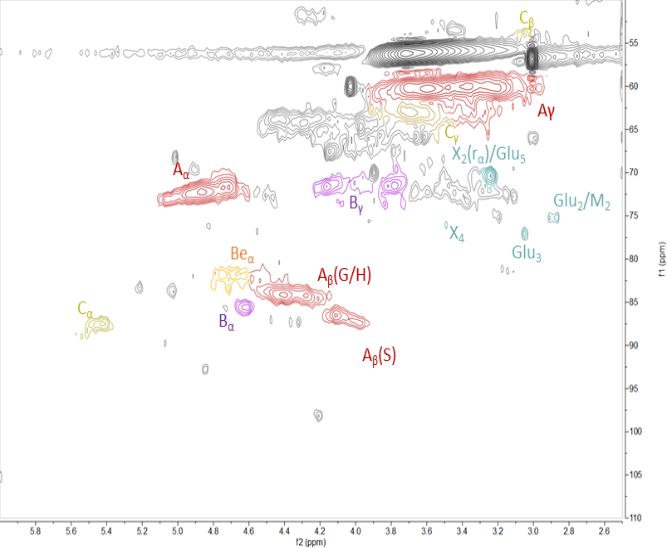

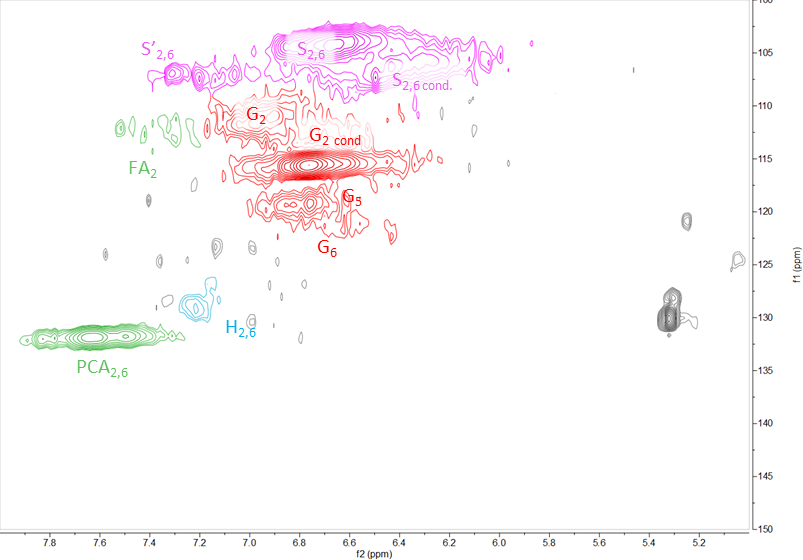

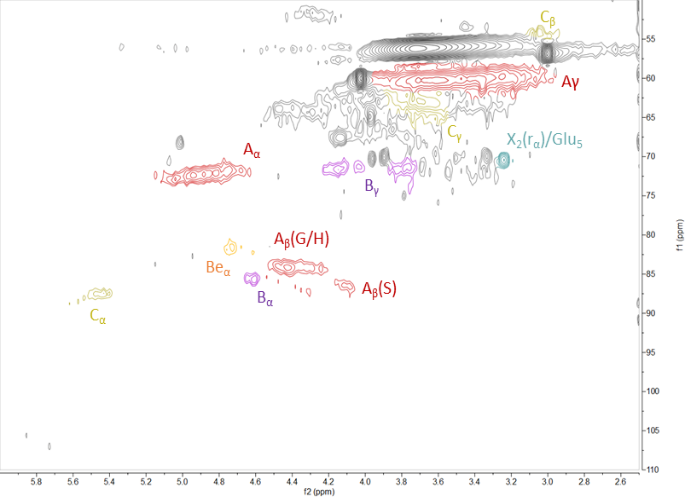

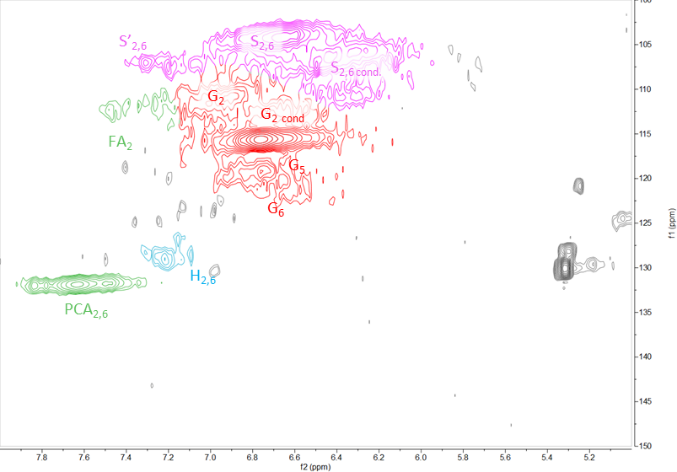

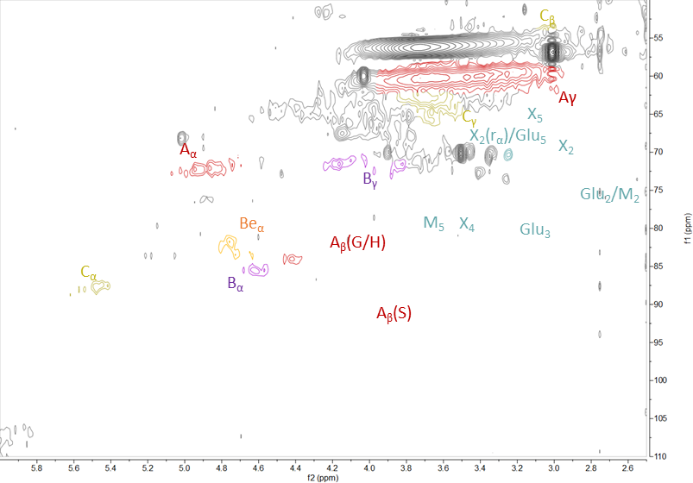

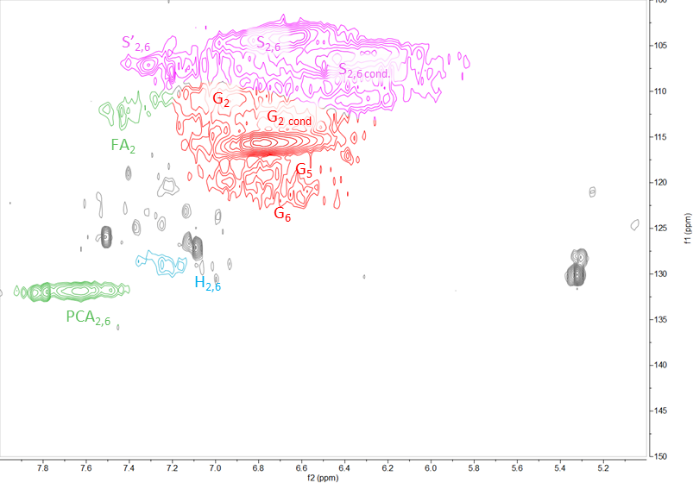


(a)

(b)

(c)

Aromatic Region

Fig. S1. 2D-HSQC NMR spectra of precipitated lignins obtained from pretreatment at 150 °C for
(a) 0.5 h, (b) 1 h and (c) 2 h.


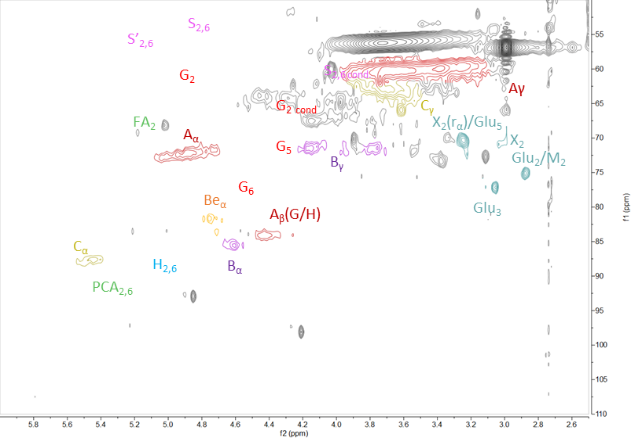

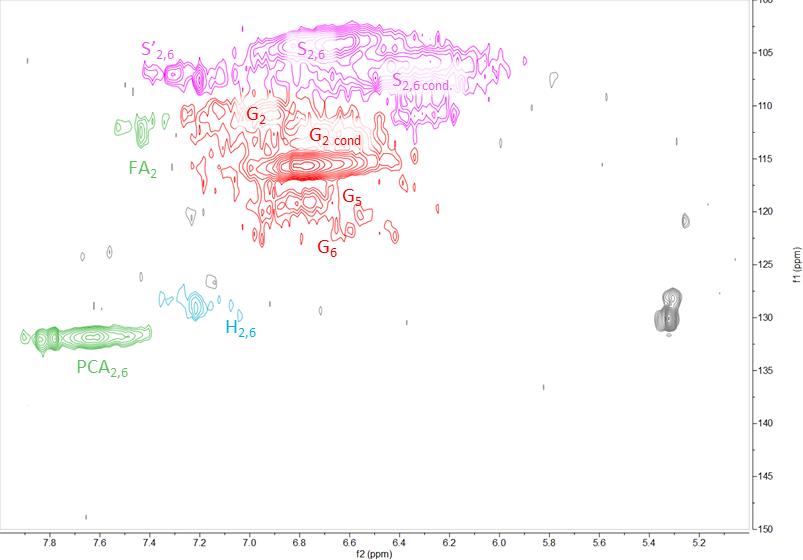

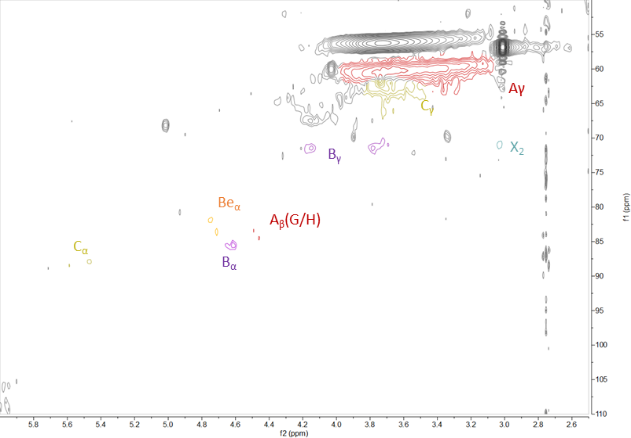

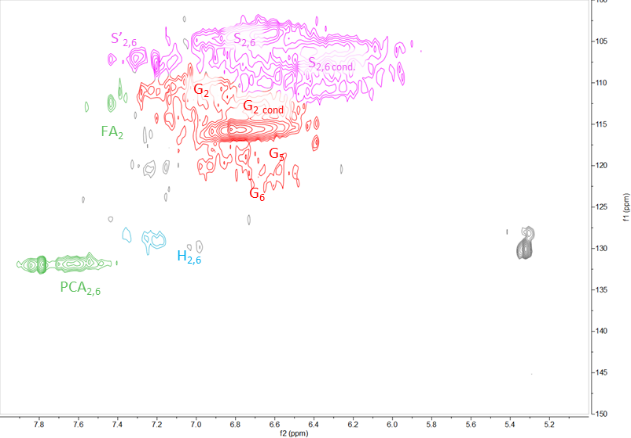

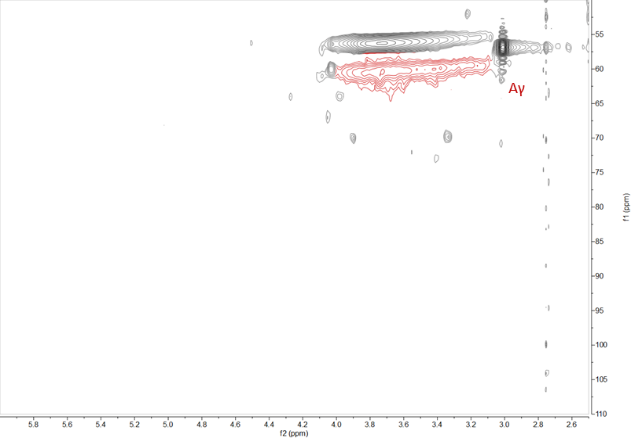

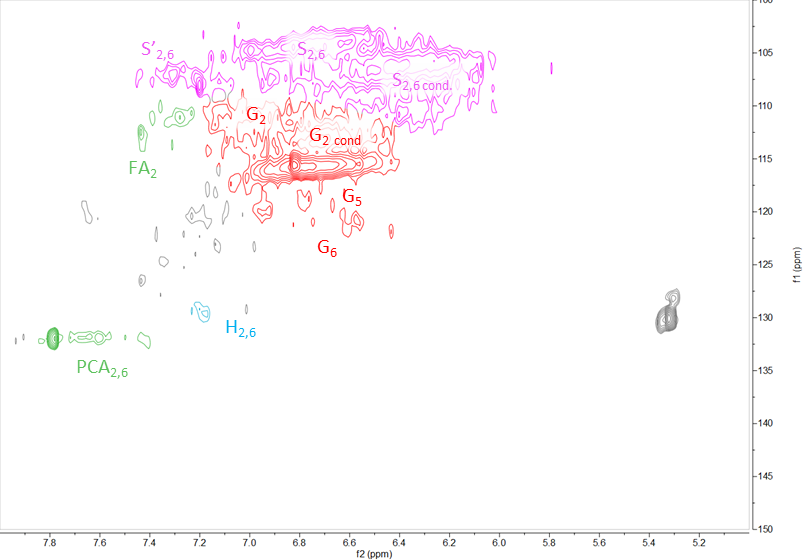


Side-Chain Region

(a)

(b)

(c)

Aromatic Region

Fig. S2. 2D-HSQC NMR spectra of precipitated lignins obtained from pretreatment at 170 °C for
(a) 0.5 h (b) 1 h and (c) 2 h.


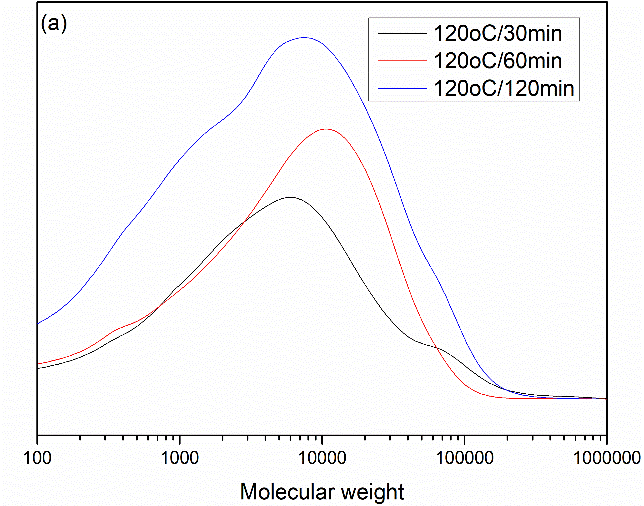

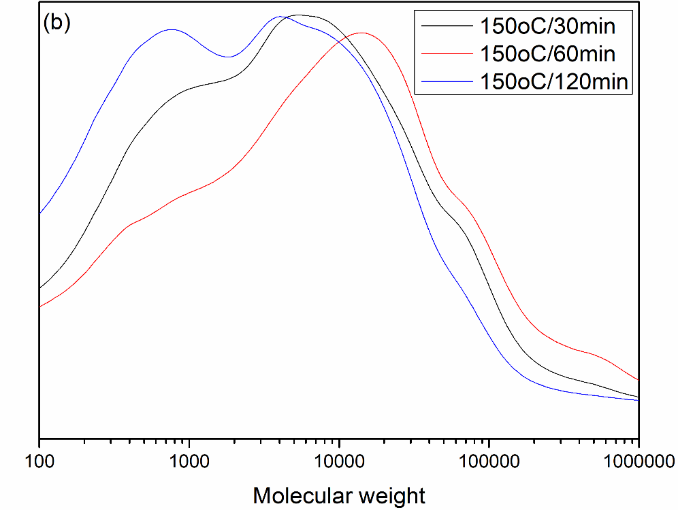

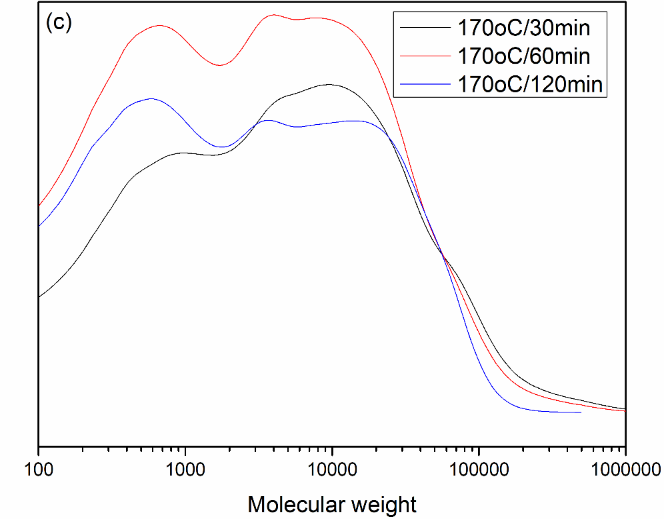


Fig. S3. GPC profiles of lignins isolated from pretreatment of OPEFB with 80wt% [DMBA][HSO_4_] and 20wt% water at a 1:10 biomass-to-solvent ratio under different conditions.
